# The modified alternative (*G*’/*G*)-expansion method to nonlinear evolution equation: application to the (1+1)-dimensional Drinfel’d-Sokolov-Wilson equation

**DOI:** 10.1186/2193-1801-2-327

**Published:** 2013-07-18

**Authors:** M Ali Akbar, Norhashidah Hj Mohd Ali, Syed Tauseef Mohyud-Din

**Affiliations:** Department of Applied Mathematics, University of Rajshahi, Rajshahi, Bangladesh; School of Mathematical Sciences, Universiti Sains Malaysia, Penang, Malaysia; Department of Mathematics, HITEC University, Taxila Cantt, Pakistan

**Keywords:** (*G*’/*G*)-expansion method, Travelling wave solutions, DSW equation, Nonlinear evolution equations, 02.30.Jr, 05.45.Yv, 02.30.Ik

## Abstract

Over the years, (*G*’/*G*)–expansion method is employed to generate traveling wave solutions to various wave equations in mathematical physics. In the present paper, the alternative (*G*’/*G*)–expansion method has been further modified by introducing the generalized Riccati equation to construct new exact solutions. In order to illustrate the novelty and advantages of this approach, the (1+1)-dimensional Drinfel’d-Sokolov-Wilson (DSW) equation is considered and abundant new exact traveling wave solutions are obtained in a uniform way. These solutions may be imperative and significant for the explanation of some practical physical phenomena. It is shown that the modified alternative (*G*’/*G*)–expansion method an efficient and advance mathematical tool for solving nonlinear partial differential equations in mathematical physics.

## Introduction

After the observation of soliton phenomena by John Scott Russell in 1834 (Wazwaz 2009) and since the KdV equation was solved by Gardner et al. ( 1967) by inverse scattering method, finding exact solutions of nonlinear evolution equations (NLEEs) has turned out to be one of the most exciting and particularly active areas of research. The appearance of solitary wave solutions in nature is quite common. Bell-shaped sech-solutions and kink-shaped tanh-solutions model wave phenomena in elastic media, plasmas, solid state physics, condensed matter physics, electrical circuits, optical fibers, chemical kinematics, fluids, bio-genetics etc. The traveling wave solutions of the KdV equation and the Boussinesq equation which describe water waves are well-known examples. Apart from their physical relevance, the closed-form solutions of NLEEs if available facilitate the numerical solvers in comparison, and aids in the stability analysis. In soliton theory, there are several techniques to deal with the problems of solitary wave solutions for NLEEs, such as, Hirota’s bilinear transformation (Hirota 1971), Backlund transformation (Rogers & Shadwick 1982), improved homotopy perturbation (Jafari & Aminataei 2010), Darboux transformation (Zhaqilao 2010), tanh-function (Malfliet 1992), homogeneous balance (Wang 1996), Jacobi elliptic function (Liu et al. 2001; Ali 2011), F-expansion (Zhou et al. 2003) and Exp-function (He & Wu 2006; Abdou et al. 2007; Akbar & Ali 2011; Naher et al. 2012). It is to be highlighted that Marinca and Herişanu ( 2011) applied a new approach for calculating a kind of explicit exact solution of nonlinear differential equations and in the simiar context obtained exact solutions of the Duffing and double-well Duffing equations. They implemented the new proposed procedure by using a quotient trigonometric function expansion method and also proved that the introduced method could be easily applied to solve other nonlinear differential equations.

Recently, Wang et al. ( 2008) established a widely used direct and concise method called the (*G*’/*G*)-expansion method for obtaining the exact travelling wave solutions of NLEEs, where *G*(*ξ*) satisfies the second order linear ordinary differential equation (ODE) *G*″ + *λ G*′ + *μG* = 0, where *λ* and *μ* are arbitrary constants. Applications of the (*G*’/*G*)-expansion method can be found in the articles (Bekir 2008; Naher et al. 2011; Akbar et al. 2012; Kol & Tabi 2011; Zayed & Gepreel 2009; Zayed 2009a; Zhang et al. 2008a; Zhang et al. 2008b; Abazari 2010; Liu et al. 2010) for better understanding.

In order to establish the effectiveness and reliability of the (*G*’/*G*)-expansion method and to expand the possibility of its application, further research has been carried out by several researchers. For instance, Zhang et al. ( 2010) presented an improved (*G*’/*G*)-expansion method to seek more general traveling wave solutions. Zayed ( 2009b) presented a new approach of the (*G*’/*G*)-expansion method where *G*(*ξ*) satisfies the Jacobi elliptic equation [*G*′(*ξ*)]^2^ = *e*_2_*G*^4^(*ξ*) + *e*_1_*G*^2^(*ξ*) + *e*_0_,  *e*_2_, *e*_1_, *e*_0_ are arbitrary constants, and obtained new exact solutions. Zayed ( 2011) again presented an alternative approach of this method in which *G*(*ξ*) satisfies the Riccati equation *G*′(*ξ*) = *A* + *B G*^2^(*ξ*), where *A* and *B* are arbitrary constants.

Still, substantial work has to be done in order for the (*G*’/*G*)-expansion method to be well established, since every nonlinear equation has its own physically significant rich structure. For finding the new exact solutions of NLEEs, it is important to present various method and ansatz, but it seems to be more important how to obtain more new exact solutions to NLEEs under the known method and ansatz. In the present article, we further modify the alternative (*G*’/*G*)-expansion method (presented by Zayed ( 2011)) by introducing the generalized Riccati equation mapping, its twenty seven solutions and constructed abundant new traveling wave solutions of the DSW equation.

## The method

Suppose the general nonlinear partial differential equation,1

where *u*=*u*(*x*,*t*) is an unknown function, *P* is a polynomial in *u*(*x*,*t*) and its partial derivatives in which the highest order partial derivatives and the nonlinear terms are involved. The main steps of the modified alternative (*G*’/*G*)-expansion method combined with the generalized Riccati equation mapping are as follows:

Step 1: The travelling wave variable ansatz2

where *V* is the speed of the traveling wave, permits us to transform the Equation (1) into an ODE:3

where the superscripts stands for the ordinary derivatives with respect to *ξ*.

Step 2: Suppose the traveling wave solution of Equation (3) can be expressed by a polynomial in (*G*’/*G*) as follows:4

where *G*’/*G*(*ξ*) satisfies the generalized Riccati equation,5

where *a*_*n*_ (*n* = 0, 1, 2, ⋯, *m*), *r*, *p* and *q* are arbitrary constants to be determined later.

The generalized Riccati Equation (5) has twenty seven solutions (Zhu, 2008) as follows:

Family 1: When *p*^2^ − 4 *q r* < 0 and *pq*≠0 (or *r q*≠0), the solutions of Equation (5) are,

where *A* and *B* are two non-zero real constants and satisfies the condition *A*^2^ − *B*^2^ > 0.

Family 2: When *p*^2^ − 4 *q r* > 0 and *pq*≠0 (or *r q*≠0), the solutions of Equation (5) are,

where *A* and *B* are two non-zero real constants and satisfies the condition *B*^2^ − *A*^2^ > 0.

Family 3: When *r*=0 and *pq*≠0, the solutions of Equation (5) are,

where *d* is an arbitrary constant.

Family 4: When *q*≠0 and *r*=*p*=0, the solution of Equation (5) is,

where *c*_1_ is an arbitrary constant.

Step 3: To determine the positive integer *m*, substitute Equation (4) along with Equation (5) into Equation (3) and then consider homogeneous balance between the highest order derivatives and the nonlinear terms appearing in Equation (3).

Step 4: Substituting Equation (4) along with Equation (5) into Equation (3) together with the value of *m* obtained in step 3, we obtain polynomials in *G*^*i*^ and *G*^*–i*^ (*i* = 0, 1, 2, 3 ⋯) and vanishing each coefficient of the resulted polynomial to zero, yields a set of algebraic equations for *a*_*n*_*p*, *q*, *r* and *V*.

Step 5: Suppose the value of the constants *a*_*n*_*p*, *q*, *r* and *V* can be determined by solving the set of algebraic equations obtained in step 4. Since the general solutions of Equation (5) are known, substituting, *a*_*n*_*p*, *q*, *r* and *V* into Equation (4), we obtain new exact traveling wave solutions of the nonlinear evolution Equation (1).

## Some new traveling wave solutions of the DSW equation

In this section, the modified alternative (*G*’/*G*)-expansion method is employed to construct some new traveling wave solutions of the (1+1)-dimensional Drinfel’d-Sokolov-Wilson (DSW) equation which is very important nonlinear evolution equation in mathematical physics and engineering and have been paid attention by many researchers. Some exact solutions of the DSW equation were found in the literature. In general, the solutions of the DSW equation have been obtained by means of an ansatz method. Included in the methods are the elliptic-function (Chen & Zhang 2003; Liu et al. 2005), Exp-function (He et al. 2010), Darboux transformation (Guo & Wu 2010), improved F-expansion (Zha & Zhi 2008), Variational iteration (Zhang 2011) and Adomian’s decomposition (Inc 2006). It is to be highlighted that Marinca et. al. ( 2011) presented quotient trigonometric function expansion method to find explicit and exact solutions to cubic Duffing and double-well Duffing equations. Moreover, a detailed study is made by Yang ( 2012) on local fractional differential equations and its Applications, Local Fractional Functional Analysis and its Applications along with local fractional variation iteration and local fractional Fourier series methods. He ( 2012) has also given a comprehensive analysis of Asymptotic methods for solitary solutions and compactons. Inspired and motivated by the ongoing research in this area, we apply the modified alternative (*G*’/*G*)-expansion method for searching its new solitary wave solutions. Let us consider the DSW equation:67

Now, we use the wave transformation Equation (2) into Equations (6) and (7), which yield:89

According to step 3, the solution of Equations (8) and (9) can be expressed by a polynomial in (*G*’/*G*) as follows:10

and11

where *a*_*i*_, (*i* = 0, 1, 2, ⋯, *m*) and *b*_*j*_, (*j* = 0, 1, 2, ⋯, *n*) all are constants to be determined and *G*’/*G*(*ξ*) satisfies the generalized Riccati Equation (5). Considering the homogeneous balance between the highest order derivatives and the nonlinear terms in Equations (8) and (9), we obtain *m*=2 and *n*=1.

Therefore, solution Equations (10) and (11) take the form respectively1213

By means of Equation (5), Equations (12) and (13) can be rewritten respectively as,14

and15

Substituting Equations (14) and (15) into Equations (8) and (9), the left hand sides of these equations are converted into polynomials in *G*^*i*^ and *G*^− *i*^, (*i* = 0, 1, 2, 3, ⋯). Setting each coefficient of these polynomials to zero, we obtain a set of simultaneous algebraic equations for *a*_0_, *a*_1_, *a*_2_, *b*_0_, *b*_1_, *p*, *q*, *r* and *V* as follows:16

Solving the over-determined set of algebraic equations by using the symbolic computation software, such as, Maple, we obtain17

where *b*_1_, *p*, *q* and *r* are arbitrary constants.

Now on the basis of the solutions of Equation (5), we obtain some new types of solutions of Equations (6) and (7).

### Family 1

When *p*^2^ − 4 *q r* < 0 and *pq*≠0 (or *r q*≠0), the periodic form solutions of Equations (6) and (7) are:

where  and *b*_1_, *p*, *q*, *r* are arbitrary constants.

where *A* and *B* are two non-zero real constants satisfies the condition *A*^2^ − *B*^2^ > 0.

### Family 2

When *p*^2^ − 4 *q r* > 0 and *pq*≠0 (or *rq*≠0), the soliton and soliton-like solutions of Equations (6) and (7) are:

where  and *b*_1_, *p*, *q*, *r* are arbitrary constants.

where *A* and *B* are two non-zero real constants and satisfies the condition *B*^2^ − *A*^2^ > 0.

### Family 3

When *r*=0 and *pq*≠0, the solutions of Equations (6) and (7) are:

### Family 4

When *q*≠0 and *r*=*p*=0, the solutions of Equations (6) and (7) are:

where *c*_1_ is an arbitrary constant.

Because of the arbitrariness of the parameters *b*_1_, *p*, *q* and *r* in the above families of solution, the physical quantities *u* and *v* might possess physically significant rich structures.

## Graphical presentation

**Figure 1:**
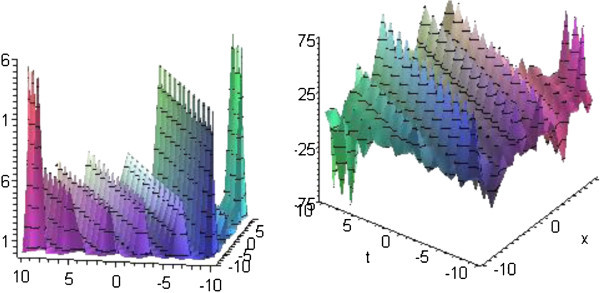
**Solitons corresponding to solutions*u***_**1**_**and*v***_**1**_**for*p*=*q*=2,*r*=3 and*b***_**1**_**=1.**

**Figure 2:**
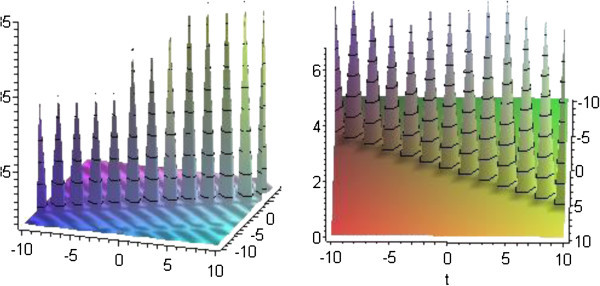
**Solitons corresponding to solutions*u***_**5**_**and*v***_**5**_**for*p*=*q*=1,*r*=2 and*b***_**1**_**=1.**

**Figure 3:**
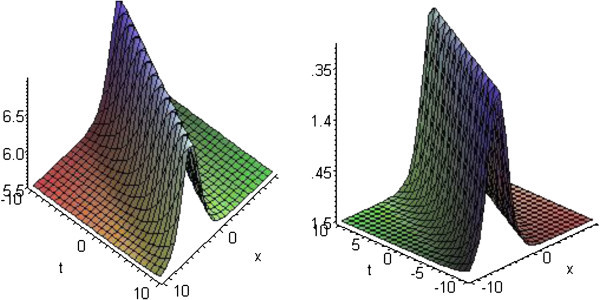
**Solitons corresponding to solutions*u***_**13**_**and*v***_**13**_**for*p*=3,*q*=2,*r*=1 and*b***_**1**_**=1.**

**Figure 4:**
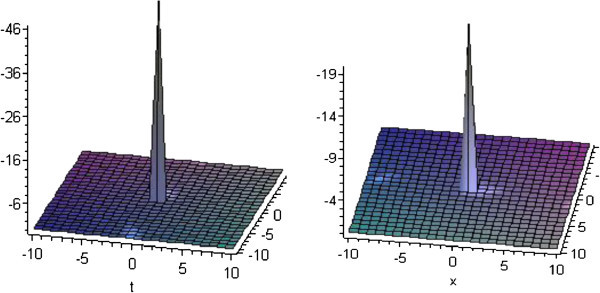
**Solitons corresponding to solutions*u***_**24**_**and*v***_**24**_**for*p*=3,*q*=2,*r*=1 and*b***_**1**_**=5.**

**Figure 5:**
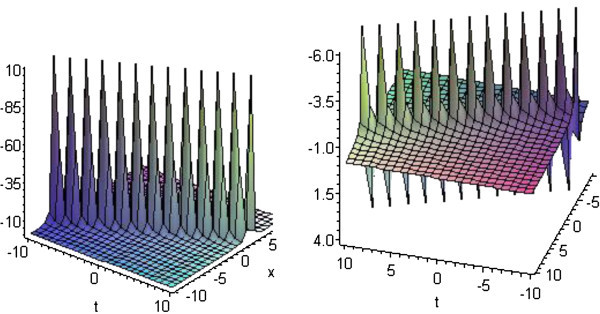
**Solitons corresponding to solutions*u***_**27**_**and*v***_**27**_**for*p*=0,*q*=1,*r*=0 and*b***_**1**_**=5 and*c***_**1**_**=1.**

Graph is a powerful tool for communication and describes lucidly the solutions of the problems. Therefore, some graphs of the solutions are given below. The graphs readily have shown the solitary wave form of the solutions (Figures 1, 2, 3, 4 and 5).

## Conclusion

In this article, the alternative (*G*’/*G*)-expansion method has been modified by introducing the generalized Riccati equation mapping and obtain abundant exact traveling wave solutions of the (1+1)-dimensional DSW equation with the help of symbolic computation. It is important to point out that the obtained solutions have not been reported in the previous literature. The new type of traveling wave solutions found in this article might have significant impact on future research. We assured the correctness of our solutions by putting them back into the original Equations (6) and (7). This article is only an imploring work and we look forward the modified alternative (*G*’/*G*)-expansion method may be applicable to other kinds of NLEEs in mathematical physics. The extension of the method proposed in this paper to solve NLEEs with variable coefficients deserves further investigations.

## References

[CR1] Abazari R (2010). The (*G*’/*G*)-expansion method for Tziteica type nonlinear evolution equations. Math Comput Modelling.

[CR2] Abdou MA, Soliman AA, Basyony ST (2007). New application of exp-function method for improved Boussinesq equation. Phys Lett A.

[CR3] Akbar MA, Ali NHM (2011). Exp-function method for duffing equation and New solutions of (2+1) dimensional dispersive long wave equations. Prog Appl Math.

[CR4] Akbar MA, Ali NHM, Zayed EME (2012). Abundant exact traveling wave solutions of the generalized Bretherton equation via the improved (G'/G)-expansion method. Commun. Theor. Phys.

[CR5] Ali AT (2011). New generalized Jacobi elliptic function rational expansion method. J Comput Appl Math.

[CR6] Bekir A (2008). Application of the (*G*’/*G*)-expansion method for nonlinear evolution equations. Phys Lett A.

[CR7] Chen HT, Zhang HQ (2003). Improved Jacobin elliptic function method and its applications. Chaos Solitons and Fract.

[CR8] Gardner CS, Greener JM, Kruskal MD (1967). Phys Rev Lett.

[CR9] Guo GX, Wu LH (2010). Darboux transformation and explicit solutions for Drinfel’d–Sokolov–Wilson Equation. Commun Theor Phys.

[CR10] He JH (2012). Asymptotic methods for solitary solutions and compactons. Abstr Appl Anal.

[CR11] He JH, Wu XH (2006). Exp-function method for nonlinear wave equations. Chaos Solitons Fract.

[CR12] He YH, Long Y, Li SL (2010). Exact solutions of the Drinfel’d-Sokolov-Wilson Equation using the F-expansion method combined with Exp-function method. Int Math Forum.

[CR13] Hirota R (1971). Exact solution of the KdV equation for multiple collisions of solitions. Phys Rev Lett.

[CR14] Inc M (2006). On numerical doubly periodic wave solutions of the coupled Drinfel’d–Sokolov–Wilson equation by the decomposition method. Appl Math Comput.

[CR15] Jafari MA, Aminataei A (2010). Improvement of the homotopy perturbation method for solving nonlinear diffusion equations. Phys Scr.

[CR16] Kol GR, Tabi CB (2011). Application of the (*G*’/*G*)-expansion method to nonlinear blood flow in large vessels. Phys. Scr.

[CR17] Liu S, Fu Z, Liu S, Zhao Q (2001). Jacobi elliptic function expansion method and periodic wave solutions of nonlinear wave equations. Phys. Lett. A.

[CR18] Liu S, Fu Z, Liu S (2005). Periodic solutions for a class of coupled nonlinear partial differential equations. Phys Lett A.

[CR19] Liu X, Tian L, Wu Y (2010). Exact solutions of the generalized Benjamin-Bona-Mahony equation. Math. Prob. Engr.

[CR20] Malfliet M (1992). Solitary wave solutions of nonlinear wave equations. Am J Phys.

[CR21] Marinca V, Herişanu N (2011). Explicit and exact solutions to cubic Duffing and double-well Duffing equations. Math Comp Model.

[CR22] Naher H, Abdullah FA, Akbar MA (2011). The (*G*’/*G*)-expansion method for abundant travelling wave solutions of Caudrey-Dodd-Gibbon equation. Math Prob Engr.

[CR23] Naher H, Abdullah FA, Akbar MA (2012). New traveling wave solutions of the higher dimensional nonlinear partial differential equation by the Exp-function method. J. Appl. Math.

[CR24] Rogers C, Shadwick WF (1982). Backlund Transformations.

[CR25] Wang ML (1996). Exact solutions for a compound KdV-Burgers equation. Phys Lett A.

[CR26] Wang ML, Li X, Zhang J (2008). The (*G*’/*G*)-expansion method and traveling wave solutions of nonlinear evolution equations in mathematical physics. Phys Lett A.

[CR27] Wazwaz MA (2009). Partial Differential Equations and Solitary Waves Theory.

[CR28] Yang XJ (2012). Advanced local fractional calculus and its applications.

[CR29] Zayed EME (2009). The (*G*’/*G*)-expansion method and its applications to some nonlinear evolution equations in the mathematical physics. J Appl Math Comput.

[CR30] Zayed EME (2009). New traveling wave solutions for higher dimensional nonlinear evolution equations using a generalized (*G*’/*G*)-expansion method. J Phys A: Math Theor.

[CR31] Zayed EME (2011). The (*G*’/*G*)-expansion method combined with the Riccati equation for finding exact solutions of nonlinear PDEs. J Appl Math Inform.

[CR32] Zayed EME, Gepreel KA (2009). The (*G*’/*G*)-expansion method for finding traveling wave solutions of nonlinear PDEs in mathematical physics. J Math Phys.

[CR33] Zha XQ, Zhi HY (2008). An Improved F-Expansion Method and its Application to Coupled Drinfel’d–Sokolov–Wilson Equation. Commun Theor Phys.

[CR34] Zhang WM (2011). Solitary solutions and singular periodic solutions of the Drinfeld-Sokolov-Wilson Equation by variational approach. Appl Math Sci.

[CR35] Zhang S, Tong J, Wang W (2008). A generalized (*G*’/*G*)-expansion method for the mKdV equation with variable coefficients. Phys Lett A.

[CR36] Zhang J, Wei X, Lu Y (2008). A generalized (*G*’/*G*)-expansion method and its applications. Phys Lett A.

[CR37] Zhang J, Jiang F, Zhao X (2010). An improved (*G*’/*G*)-expansion method for solving nonlinear evolution equations. Int J Comput Math.

[CR38] Zhaqilao (2010). Darboux transformation and multi-soliton solutions for some (2+1)-dimensional nonlinear equations. Phys Scr.

[CR39] Zhou YB, Wang ML, Wang YM (2003). Periodic wave solutions to coupled KdV equations with variable coefficients. Phys Lett A.

[CR40] Zhu S (2008). The generalized Riccati equation mapping method in nonlinear evolution equation: application to (2+1)-dimensional Boiti-Leon-Pempinelle equation. Chaos Soliton and Fract.

